# Correspondence

**Published:** 1869-04

**Authors:** 


### Correspondence.

For the Buffalo Medical and Surgical Journal.

*Dear Doctor:*—I send you a statement of a remarkable case of paralysis, (peraplégia) drawn up by the patient himself, an intelligent, self-educated man, who died several years ago, not long after the description was written. The physician who made the *post mortem* examination, (the late Prof. T. Childs, M. D.,) informed me that the spinal cord was completely disorganized at the point of injury, the lower dorsal and upper lumbar vertebra, so that no nervous influence could have been transmitted to the parts below the seat of the injury. (There was perfect dislocation of the eleventh vertebra.) So far as I know, the case has never been published. I take the liberty of condensing the history somewhat, but without changing the sense in the slightest degree. If you think it worthy of publication, it is much at your service. L.

"To Dr. S. J.:

"Dear Sir:—At your request I am induced to make a statement of some of the effects produced on my system by a painful accident which occurred some *forty* years since, which you seem to think might prove interesting and perhaps useful to the medical profession. I was born in 1782, and was apprenticed to the house building business. On the sixth day of November, 1804, I fell from a scaffold forty feet to the ground, without meeting anything to break the fall. I had no recollection of anything that transpired after the scaffold gave way until I was carried into the house, and the doctor was preparing to open a vein in my arm. The shock was so great that I lay mostly unconscious for several hours. I was believed, however, by those present to be *dead* below the hips. The next morning I was carried on a litter to my father's, about a mile distant, where I was examined by several physicians. When examined I was placed across the bed, with my face downward, and one stout man took hold under my arms, another around my feet, and with their knees braced against the bed-post they pulled lustily, while the surgeons with their fingers felt along the spine to see if they could find anything out of the way, and at one place (at the joint of the lumbar vertebra) they thought at first they discovered a slight depression, but on stripping my brother and placing him in a similar position, they could not be satisfied that there was any difference. There was no fracture nor dislocation apparently of any bones; not a scratch nor even a place where the color of the skin was changed. I apprehend that I must have struck upon the lower part of the back and hips, and as it was a cold day I had an increased quantity of clothes upon me, and these fastened snug and tight, and this I think most likely saved my life, for although no visible or tangible mark could be discovered, yet the whole system below the supposed point of injury, was completely paralyzed, both externally and internally, and has remained so till this day, *a space of forty-four years!* Although there has been some little alteration from the supposed point of injury down to the knee, yet below this no improvement whatever has taken place. I have frequently cut, burned, bruised and injured my legs in various ways without knowing it, until I discovered the wound by sight.

Some fourteen years ago I fell and broke both bones of the right leg, three or four inches below the knee, and knew nothing of the break until I attempted to get up, when I found it to swing round like a leathern strap, instead of keeping its place. I immediately got into my bed, sent for a surgeon, had it dressed, splintered, bandaged, etc., in the usual manner, and after lying upon my back the usual time, I again got to my crutches and walked about without having experienced during the whole time the least sensation in it more than in the crutch I walked upon.

The day after I received the injury a large *trough* was prepared, and for ten or twelve days I was daily put into a warm bath, and smart friction with a dozen hands was applied for a considerable time, but without any apparent benefit, except to remove the extreme soreness with which I was afflicted, after so severe a wrench from the fall. Electricity, it was thought, might be advantageous, and an apparatus was provided, my bed was placed upon glass supporters, and I was frequently insulated and remained for a long time so filled with the fluid that sparks would fly off from the surface on the approach of any substance not a non-conductor. Slight shocks were likewise made to pass across the line of demarcation, where sensibility became extinct. A galvanic battery was likewise procured and shocks passed in the same manner, and although they were so frequent and powerful as to produce blisters, I do not know that I received any benefit from the application. My evacuations were, of course, entirely suppressed, but after making use of the catheter for three or four weeks, I was enabled by making great exertion, to force off my water in the usual manner, and for many years I had but little trouble from that source, unless I neglected it so long or made violent exertions, in which case it would pass off involuntarily. For many years past my greatest difficulty has been an almost entire inability to retain it at all. With the fæces I have much more trouble, for as the sphincter of the anus was entirely paralyzed, I would do nothing by injections, and hence being obliged to take cathartics so frequently, it soon became necessary to increase the dose greatly, which was reducing me quite rapidly. In this dilemma, searching for some relief, I introduced my finger into the rectum, and found hard balls of fecal matter, filling it and pressing upon the rectum.

By the use of the finger and by exerting, at the same time, the muscles of the abdomen two or three times a day, enough was accomplished to keep me comfortable for twelve or fourteen years. This was my invariable practice when I retired to bed and when I arose in the morning. The matter discharged was as dry and hard as the small balls that pass from sheep, and might be handled without soiling the hands. It has since become less dry and hard, and I can generally force it away by natural exertion, though I have occasion even now to have recourse to the old practice. It is, however, never so loose as to soil my clothes, unless when I have diarrhœa, which is very seldom.

I have said that the injury was supposed to be at the second joint of the lumbar region, and from that down the back, seat, scrotum, and under side of the thighs; sensation has never returned. At the time of the injury, this insensibility passed nearly straight around me, but I have perceived that sensation was gradually passing down in front. This, at first, was quite considerable, and continued, though with less and less progress, for nearly a year, when it had reached to within two or three inches of the knees. The sensation was nearly perfect upon the abdomen, and I had acquired so much power over the anterior muscles of the thigh, that I could move and raise and draw up my legs upon the bed; whereas at first I could not even move them as I lay, but they appeared perfectly paralyzed, as much so as those upon the inner side, which were then and still remain perfectly senseless and inactive. The sensation upon the abdomen I think quite restored externally to its former state, even to the groin, as well as to the root of the penis and scrotum, from which it extends outwards a little. I have been tapped three times upon the left side for hydrocele. The first time when the trochar was introduced I felt a sharp stinging sensation; the two last operations were performed by myself with a sharp pointed knife, and I was careful to get as near the seam in the center as I could with safety, and I was so fortunate as to get entirely beyond all sensation, and did the work well without producing the least particle of pain whatever.

My situation, internally, I am not so well able to describe. The urethra is entirely paralyzed, and on inserting the catheter I feel nothing of it until it reaches the prostrate gland. There seems to be considerable sensation there, and I should think that the bladder was somewhat affected, though the sensation is not by any means entirely destroyed, as I find the same uneasy feeling and desire to void my urine when I have retained it too long that I used formerly to feel, or nearly so. How far the numbness extends up the rectum, I am not able to say; it is total as far up as the injection pipe passes. Crowding upon it, however, produces pain in the parts adjacent, although I cannot feel the pipe itself; and when in pain from the operation of physic or the passage of urine, the pain keeps growing lower and lower till it seems to be very near the *anus*, and stops a very short time before the wind escapes.

With regard to my sexual feelings, I think my situation quite peculiar; for while the penis is entirely void of sensation, the testicles appear to be but very little affected, and perform their office with more energy, (if possible,) than before I was hurt, and quite maintain their ground in supporting the functions of the organ of amativeness, which in my system seems to be largely developed, and I have thought that conversation, amusement, sport and diversion with the other sex, so far as decency and propriety would possibly permit, have done more towards building me up, preserving my health and restoring me to activity, than all the other things put together that have been done for me. To sit in a chair, with my feet down, has always been painful to me, while to have them held on the lap and receive that gentle motion that a mother gives when *patting her baby*, produces the most agreeable effect that I can be made to experience; *although entirely insensible to the touch, externally.* Yet this sudden and gentle jar produces the most delightful sensation imaginable; and I can hardly make myself believe it possible that sensation, and that too of the most pleasant and agreeable nature has not returned. This was generally known among my friends, and many of our most virtuous and respectable females granted me this indulgence for hours, and I shall have reason to remember with gratitude while I live, the benefit which I received in this manner from some relatives at that time residing in the family, and without which I very much doubt whether I ever should have walked, as after this indulgence I always felt cheered, strengthened and invigorated; while by tilting with them on the floor, a contrary effect was produced. The same process has always the same effect, even to the present day.

On my first attempt to walk on crutches, I was supported by one person on each side, and after I had practiced awhile, I learned to balance myself a little; I tried with but one, and eventually I hastened to walk and ride, and from having a little knowledge of the *painting and profile-setting* business, I spent a number of years riding about the country in this employment; but finally my health was giving way under it, and from the awkward and embarrassing situation in which in consequence of my infirmities I frequently found myself among strangers, I was led earnestly to seek for some employment more retired and at home. The shoemaking business offered many inducements, but my feet and legs being entirely paralyzed, how should I hold my work? I at last constructed an apparatus for that purpose, and with joy relinquished my peregrination, and at the age of thirty-six apprenticed myself to the shoemaking business. In a short time I married; soon after which my wife presented me a son; again in 1823, a daughter; in 1826, another son; in 1828, a pair of twins, a son and daughter; and in 1831, my age being forty-nine and my wife forty-three, she presented me with another daughter, which was the last. I had always felt a little fearful that my infirmities might possibly attach to some of my children, but the first five were all smart, healthy and active as others, but the last was more feeble, and from many circumstances I felt quite sure that her lower limbs were equally destitute of muscular action as my own. She died at the age of nineteen months, and I very much regret that I never attempted to ascertain whether they were possessed of any sense of feeling. She would lie in her cradle and raise and throw them about as I could my own, but she never bore an ounce weight upon them, and I do not believe, if she had lived, that she ever would have walked. I think her's were exactly like my own, as she would make the same movements in bed that I could.

You will probably ask, "from whence came all these children?" I reply that from reasons perfectly satisfactory to myself, I have not the least doubt of their legitimacy. It is true the *penis* was paralyzed and all sensation destroyed; but nevertheless, the same causes that before my injury would produce an erection would produce the same effect now. I have now seen sixty-six years, and am at times a good deal troubled with asthma, I find that my sight and strength and hearing are all rapidly failing; yet I think that should I ever be married again I should not be found altogether unable to perform family duties.

I ought, perhaps, to have remarked, that while passion, imagination, inclination and instinct are all wrought up to the highest possible pitch, there is nevertheless a want of sensibility and of feeling which I formerly possessed, but which now I cannot reach, and although sexual congress has never been attended with any local feeling of pleasure, or sensation of any kind, it has often been a puzzling question in my mind why, under such circumstances, I should experience the same violent passions and sexual desires as formerly? B."

[The name of the patient is omitted, but the facts are literally as stated above, and within my knowledge.—L.]

